# A novel method to assess collagen architecture in skin

**DOI:** 10.1186/1471-2105-14-260

**Published:** 2013-08-26

**Authors:** Osman S Osman, Joanne L Selway, Parvathy E Harikumar, Claire J Stocker, Edward T Wargent, Michael A Cawthorne, Sabah Jassim, Kenneth Langlands

**Affiliations:** 1 The Clore Laboratory, The University of Buckingham, Hunter Street, Buckingham MK18 1EG, UK; 2 Department of Applied Computing, The University of Buckingham, Hunter Street, Buckingham MK18 1EG, UK

**Keywords:** Fast fourier transform, Gabor filter, Dermis, Ageing, Histology, Collagen, Basketweave, Type 2 diabetes mellitus

## Abstract

**Background:**

Texture within biological specimens may reveal critical insights, while being very difficult to quantify. This is a particular problem in histological analysis. For example, cross-polar images of picrosirius stained skin reveal exquisite structure, allowing changes in the basketweave conformation of healthy collagen to be assessed. Existing techniques measure gross pathological changes, such as fibrosis, but are not sufficiently sensitive to detect more subtle and progressive pathological changes in the dermis, such as those seen in ageing. Moreover, screening methods for cutaneous therapeutics require accurate, unsupervised and high-throughput image analysis techniques.

**Results:**

By analyzing spectra of images post Gabor filtering and Fast Fourier Transform, we were able to measure subtle changes in collagen fibre orientation intractable to existing techniques. We detected the progressive loss of collagen basketweave structure in a series of chronologically aged skin samples, as well as in skin derived from a model of type 2 diabetes mellitus.

**Conclusions:**

We describe a novel bioimaging approach with implications for the evaluation of pathology in a broader range of biological situations.

## Background

The skin is composed of three principle layers; an outer protective epidermal barrier comprised largely of keratinocytes; a deeper layer of connective tissue dermis that confers strength and elasticity; and an underlying energy store, the sub-cuticular fat. The dermis is primarily composed of extracellular matrix (ECM) proteins, assembled into a meshwork of primarily collagen fibres [1]. To date, 28 different types of collagen are known, which are categorized in eight subfamilies based on function, assembly and domain homology [2,3]. Fibrillar collagens are the major ECM component, with collagen I and IV being the predominant dermal constituents [4]. The dermis may be further subdivided into two discrete reticular and papillary layers. The reticular dermis consists of large, mature, well-organised collagen fibres in the lower layer of the dermis, interfacing with the subcutaneous fat. The papillary dermis is adjacent to the basement membrane, with thinner collagen fibres and a distinct collagen organisation [5].

In a healthy state, dermal collagen forms a ‘basketweave’ structure, with perpendicular collagen fibres intersecting at approximately 90° angles [6,7]. This basketweave structure provides textural information regarding the skin and as a loss of collagen organisation characterises many different physiological situations, including ageing [8,9], diabetes [10], scarring [11-13], fibrosis [14] and more specific diseases of connective tissue, such as Ehlers-Danlos syndrome [15], thus providing methods to assess texture in biological images has direct applications within dermatological research.

The ability to quantify collagen conformation in health and disease has important consequences for basic research, drug development and clinical diagnosis. For example, techniques facilitating accurate measurement of photo-damage, scarring, wound repair or other age-related damage are of value in determining the effectiveness of collagen repair therapies. Histological stains, such as picrosirius, effectively identify collagen in tissue specimens, and one may make qualitative assessments of ECM integrity from photomicrographs. However, unbiased image analysis methods are preferable. Segmentation-based methods have been described to assess collagen bundle thickness and orientation, although a degree of user intervention is required [16]. More sophisticated unbiased methods exploit frequency domain transformation methods, or power spectral analysis tools.

Power spectral analysis estimates the power/energy variation of an image in different frequency sub-ranges, and is directly related to the autocorrelation of an image in that it describes how closely related two points in an image are as a function of their distance and orientation. The Fourier Transform, in particular the Fast Fourier Transform (FFT), has been used to estimate the power spectrum of images, and this approach was reported by several groups in measurements of bundle thickness and spacing, as well as collagen fibre orientation [9,11,12,14,17-19].

One particular variation of this approach, the Fourier zeroth-order maximum analysis, has been used to measure the orientation of collagen fibres [14]. This method was extensively applied to a variety of different clinical situations, including identifying fibrosis in scleroderma [14], evaluating new treatments for hypertrophic scars and keloids [11,12], and assessing the effectiveness of dermal substitutes in clinical trials [18]. Initial attempts to utilise first-order maximum Fourier analysis required substantial observer input [14] but this approach was refined so that the user simply selected the area of interest for analysis and a measure of collagen orientation was calculated by determining stretch or elongation of the FFT spectrum [19]. In this way, differences in bundle thickness, spacing and orientation in scar tissue compared to normal skin were measured.

Although the methods described above identify gross collagen changes associated with pathological states, they are not sufficiently sensitive to measure incremental changes in architecture seen in, for example, the progressive loss of basketweave with chronological age. A method to facilitate the quantification of textural information is, therefore, required. Gabor filters are known for their similarity to human visual system (HVS) models in interpreting image texture as they provide a multi-channel bank of filters capable of analysing images at different narrow spatial frequencies and orientations. The link between HVS and Gabor filters was established by the pioneering work of Daugman on image analysis/compression and iris recognition [20,21]. It has since been successfully used for texture representation, segmentation and discrimination. Therefore, we sought to build on these published methods by combining Gabor filter and Fourier transform techniques to measure collagen fibre orientation in a series of images derived from picrosirius-stained mouse skin. Polarised light microscopy clearly reveals the basketweave structure of the dermis [22] and by initially applying a Gabor filter in eight angles to our images before creating a Fourier spectrum, we can generate a metric for collagen structure, indicating the integrity of the collagen basketweave. This provides increased sensitivity and decreased user input which is prone to human error and bias. To test the analysis platform we have measured the effect of chronological ageing in wild-type (WT) mice and assessed dermal integrity in a mouse model of type 2 diabetes. The improved performance of the Gabor analysis in mouse skin, which is notoriously difficult to analyze compared to human skin, confirms the superior nature of this platform for dermal structure analysis.

## Methods

### Biological methods

#### Animal models

All procedures were conducted in accordance with the UK Government Animals (Scientific Procedures) Act 1986 and approved by the University of Buckingham Ethical Review Board. C57Bl6, type 2 diabetic *Lepr*^*db*^*/Lepr*^*db*^ (*db/db*) mice on the C57BLKS/J background and control C57BLKS/J (Misty) mice were maintained on chow diets fed *ad libitum* under standard conditions (BeeKay Number 1, B&K Universal Ltd, Leeds, UK). Mice were obtained from Charles River (Manston, UK) aged 5-6wk. Wild-type C57 mice were killed at 3mth, 8mth, 12mth and 20mth of age and Misty and *db/db* mice were killed at 6wk, 3mth, 5mth, and 6mth of age. By 12wk *db/db* animals were hyperglycaemic and a meaningful model of human type II diabetes. Freely fed males were used for all studies, and tissues from at least 3 animals per group were studied.

#### Tissue

Once animals were euthanized, dorsal skin biopsies were taken immediately and snap frozen in liquid nitrogen prior to storage at −80°C until all samples were ready for simultaneous processing to minimise artefacts. Samples were transferred into cold (4°C) 10% neutral buffered formalin, then fixed for 7-8 h at room temperature. This was followed by dehydration, clearing and wax immersion in an automated tissue processor as standard. Rectangular pieces of skin were placed on their sides in moulds such that sections would be cut orthogonal to the epidermal surface, before embedding in paraffin wax. 4 μm thick sections were cut using a rotary microtome with a knife angle of 35° and a clearance angle between 1° and 5°, before transfer to positively-charged glass slides. Haematoxylin and Eosin (H&E) staining was carried out as standard to confirm tissue integrity and orientation in all samples.

#### Staining

**Figure 1:**
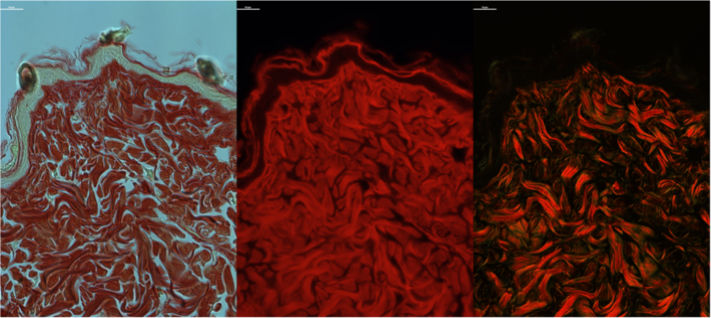
**Picrosirius stained mouse skin.** C57Bl6 mouse skin stained with picrosirius and viewed under bright-field (left panel), fluorescence (abs/em ~581/644 nm, centre panel) or dark-field cross-polar optics (right panel, original magnification 90X, scale bar = 10 μm).

Collagen organisation was studied using a picrosirius method as described previously [22-24]. Briefly, slides were stained for 1 hour at room temperature in 0.1% Direct Red 80 in saturated picric acid prior to differentiation in 0.5% acetic acid, dehydration, clearing and mounting. Slides were imaged in bright-field, dark-field and under fluorescence with a Nikon Eclipse Ti-E inverted microscope equipped with cross-polar optics (Nikon, Kingston, UK) and a QImaging CCD camera coupled to Nikon NIS Elements software (version 4.10.01). Images from each slide were captured at 90X magnification in at least 3 different locations per animal and averaged to provide a mean value for each animal. Auto-fluorescent and bright-field images revealed the gross structure of collagen in the dermis (Figure 1) whereas dark-field (crossed-polar microscopy) imaging of picrosirius stained skin revealed the basketweave structure of dermal collagens (Figure 1). Qualitatively, relaxation of the basketweave was seen in ageing and in pathological states [8-10].

#### Statistical analysis

Data analysis was performed using GraphPad Prism 5.0 (GraphPad Software Inc, La Jolla, CA, USA). As the data exhibited a normal distribution (as determined by the D’Aostino and Pearson omnibus normality test), two-group tests, between *db/db* and Misty or papillary and reticular compartments, were carried out using Students’ t-test, otherwise one-way ANOVAs followed by Dunnett’s post-hoc analysis where the ANOVA demonstrated significance were performed. Where appropriate, Pearson’s Correlation analysis was performed (p < 0.05). For all tests: * p < 0.05; ** p < 0.01; *** p < 0.001; ****p < 0.0001.

### Computational method development

#### Fourier transformation of skin images

Image processing was performed using MATLAB R2011a (Mathworks, Cambridge, UK) with the Image Processing Toolbox. The Fast Fourier Transformation (FFT) is an efficient algorithm to compute the Discrete Fourier transform (DFT) of a signal and its inverse. The DFT of an image extracts the strength of the different frequency waveforms contributing to the pixel values of the entire image. The DFT of an image f for any frequency pair (u,v) is a complex number that depends on all the spatial pixel values f(x,y) computed by the formula:

(1) $$f\left(u,v\right)=\frac{1}{\mathrm{MN}}\sum_{X=0}^{M‒1}\sum_{Y=0}^{N‒1}f\left(x,y\right)e^{‒j2\pi \left(\frac{\mathrm{ux}}{M}+\frac{\mathrm{vy}}{N}\right)}$$

**Figure 2:**
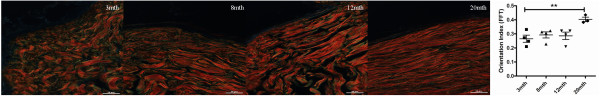
**FFT Analysis of chronological skin ageing.** Representative images of 3mth, 8mth, 12mth and 20mth C57Bl6 mouse skin stained with picrosirius and viewed under cross-polar optics (original magnification 90X; scale bar = 20 μm). The graph shows the collagen orientation index derived from the FFT, the bar equates to the mean and hair lines are standard error of the mean (S.E.M.). Statistical comparisons were achieved by comparing values via one way ANOVA with post-hoc Dunnett’s test compared to 3mth animals, n > 3 animals per group.

The amplitude (modulus) of the DFT values for all pairs of frequencies form the overall Fourier spectrum of the image. The image features/patterns in the spectrum is used to determine the relative organisation or directionality of the original image texture. Power spectral analysis of an image can be interpreted as an averaging of the FFT spectrum at different frequency sub-bands. The DFT was computed with the FFT function in MATLAB, and applied to cross-polar collagen images. Elliptical measurements of the scatter pattern were made for each spectrum, from which a collagen orientation index was generated as described in equation 3. We investigated the reported qualitative decline in dermal integrity using an implementation of FFT similar to that previously described for the evaluation of gross collagen perturbation. In this way, we were able to measure structural changes discriminating young (3mth) and old (20mth) dermis (Figure 2). However, we were not able to detect age-related changes prior to this time-point, thus a more sensitive approach was required.

#### Fourier transformation with Gabor filtering

FFTs cannot provide simultaneous information about the spatial and frequency content of an image, i.e. while FFTs can reveal the frequency content of an image, it does not localise the different frequencies. Image texture analysis requires multi-channel transforms that analyse an image at different spatial frequencies and orientation. The orientation and direction parameterized family of Gabor filters provides such a multi-channel/multi-resolution tool capable of representing both the spatial and frequency information contained in an image. A Gabor function in the spatial domain is a sinusoidal modulated Gaussian. For a 2-D Gaussian curve with a spread of σ_x_ and σ_y_ in the x and y directions, respectively, and a modulating frequency ω, the real impulse response of the filter is given by:

(2) $$g\left(x,y,\omega \right)=\frac{1}{2\pi \;\sigma_{x}\sigma_{y}}e^{‒\frac{1}{2}\left(\frac{x^{2}}{\sigma_{x}{}^{2}}+\frac{y^{2}}{\sigma_{y}{}^{2}}\right)}\cos\left(2\mathrm{\pi \omega x}\right)$$

The imaginary impulse response of the filter has a similar formula, but the cosine is replaced with the sine function. The FFT of such a Gabor function is two-shifted Gaussians at the location of the modulating frequency ω (for more detail see [21]). These properties inform the combined use of a family of Gabor filters parameterised in eight directions, followed by FFTs to investigate the quantification of distortion in the basketweave pattern of collagen structure seen in ageing or pathological states.

**Figure 3:**
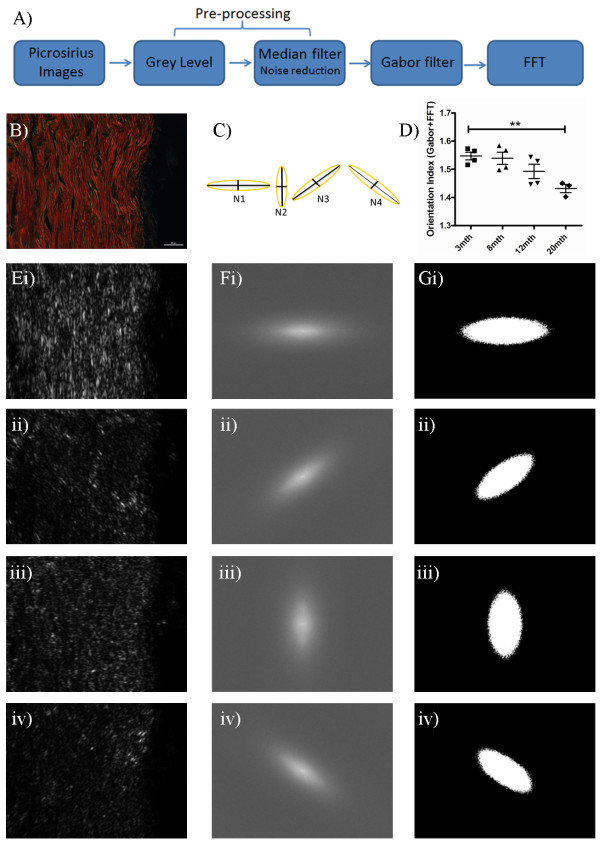
**Quantification of dermal integrity from spectra derived from Gabor filtering and FFT. A)** Flowchart depicting the different stages of analysis. **B)** Typical 3mth C57Bl6 mouse skin stained with picrosirius and viewed under cross-polar optics (original magnification 90X, scale bar = 20 μm). **C)** Illustration of the ellipse measurements generated to produce the collagen orientation index (N) from the elliptical axes generated from ω values N1-N4. **D)** Alterations in dermal integrity with age. To illustrate the procedure for quantifying spectra, the original image after pre-processing and Gabor filtering is shown in **E** (i-iv in each ω direction), and the FFT spectra in **F** (i-iv in each ω direction) conversion of the resultant spectra to binary ellipses for quantification in **G** (i-iv in each ω direction).

The key steps of the combined Gabor and FFT method are summarised in the flow diagram in Figure 3A. A representative cross-polar image of mouse skin stained with picrosirius is shown in Figure 3B. Images were converted to monochrome grey scale and a 3x3 median filter applied to remove photon noise generated during image acquisition. An 8-directional Gabor filter was applied on the input Image (x,y) using ω values of 45° + 225°, 90° + 270°, 135° + 315° and 0° + 180° to detect and highlight collagen fibre edges. Figure 3C demonstrates the superimposition of the images created by the different eight directional filters. To improve quantification, windowing was performed on the Gabor-filtered images prior to Fourier transformation to remove the vertical and horizontal lines that appear in the frequency domain as a result of the vertical and horizontal discontinuities at the edge of a standard image. Measurements derived from the elliptical shape of the scatter pattern in each of eight angles were used to quantify collagen basketweave integrity in ageing states (Figure 3D). As an example, FFT spectra derived from B) in each orientation are shown in Figure 3E-G. For each power spectrum, we generated a collagen orientation index from the resultant ellipse. Each ellipse is quantified as a function (Nωn) of the length of its minor and major axes (Figure 3F and equation 3). In order to generate an overall collagen orientation index, the ratios of maximum and minimum Nωn values are calculated for each image (Equation 4).

(3) $$\mathrm{N\omega n}=1-\frac{\mathrm{short}\;\mathrm{axis}}{\mathrm{long}\;\mathrm{axis}}$$

(4) $$N\left(\mathrm{Orientation}\;\mathrm{index}\right)=\frac{\mathrm{Max}\;\mathrm{N\omega n}}{\mathrm{Min}\;\mathrm{N\omega n}}$$

This generates a quantitative measure of basketweave integrity. If the ellipses in all directions are equal there is less order to the basketweave and equation 4 will result in a lower value, closer to 1. However, if the basketweave is intact, there will be a disproportionate amount of collagen in the 45° + 225°, and the 135° + 315° ellipses. This will result in large differences in the Nωn values and a resulting larger orientation index value (N).

## Results

### Investigation of ageing skin

By quantifying the spectra resulting from FFT images, either with or without an eight directional Gabor filter, we sought to evaluate the ability of our methodology to provide an index of collagen organisation. The collagen basketweave is known to relax from middle age in humans, and *in vivo* analysis of collagen orientation in murine skin via multi-photon confocal microscopy demonstrated measureable alterations in collagen structure from 6-12mth [9]. Furthermore, age-related decreases in collagen content, ECM fibre cross-linking, and dermal depth measurements are also detectable by 12mth [25-27].

We assessed collagen structure in skin samples prepared from mice of increasing age. Superficially, a loss of basketweave is apparent by 20mth of age (Figure 2A), and this difference could be determined in FFT images with or without Gabor filtering (Figures 2 and 3D). However, FFT alone did not identify a progressive decline in integrity from 8mth as one would have anticipated from published observations of ageing skin structure [8,10], rather a trend was only detectable with the introduction of the Gabor filtering step (Figure 3D). We were, however, able to demonstrate a significant inverse correlation between time and collagen organisation (Table 1). When compared to the use of FFT alone, the correlation between age and collagen organisation increased with Gabor filtering, with R^2^ values of 0.842 and 0.95 respectively.

**Table 1:** Correlation of ageing with collagen structure

	**All gabor**	**Papillary gabor**	**Reticular gabor**	**All FFT**	**Papillary FFT**	**Reticular FFT**
95% CI	−0.999 to −0.209	−0.999 to 0.284	−0.999 to −0.276	−0.370 to 0.999	−0.574 to 0.997	−0.171 to 0.999
P value	0.0128	0.0206	0.0111	0.0413	0.0683	0.0272
R^2^	0.950	0.920	0.956	0.842	0.7454	0.8941

**Figure 4:**
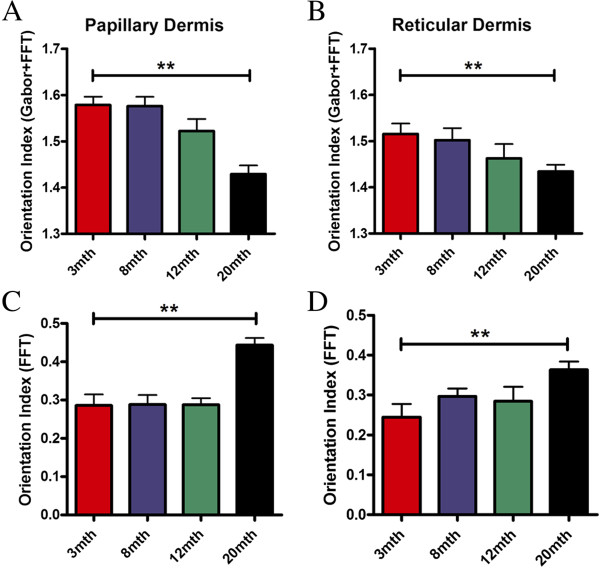
**Increasing age corresponds with differential patterns of decline in collagen organisation in the different layers of the dermis.** A progressive decline in integrity was seen in both papillary and reticular compartments by our method **(A** and **B)**. While FFT alone was able to detect changes by 20mth, there was no evidence of progressive decline in either compartment **(C** and **D)**. The bar equates to the mean and hair lines are standard error of the mean (S.E.M.). One way ANOVA with post-hoc Dunnett’s test compared to 3mth group was performed, for all tests: ** p < 0.01 (n > 3 animals per group).

The dermis is divided into superficial papillary and deeper reticular layers, distinguishable by collagen organisation. We went on to evaluate differences in age-related changes in these two compartments. The reticular dermis consistently exhibited a lower orientation index in the presence or absence of the Gabor filter (Figure 4), and while this difference did not achieve significance (p = 0.07), it is suggestive of a higher level of organisation in the papillary dermis. While FFT alone was able to detect changes by 20mth, there was no evidence of progressive decline in either compartment (Figure 4C and 4D). More importantly, use of a Gabor filter revealed differential rates of collagen basketweave degradation in the two layers, with a progressive decline in integrity in both papillary and reticular compartments using the Gabor filter (Figure 4A and B). The papillary dermis maintained consistent collagen organisation until 8mth, with a reduction in mean integrity by 12mth that was exacerbated by 20mth (Figure 4A). A more progressive loss of structure was seen in the reticular compartment, with changes between 8mth and 20mth being less marked. A significant change in reticular collagen structure between 3mth and 20mth was observed with the FFT alone (p = 0.039) and with the inclusion of the Gabor filter (p = 0.039) by unpaired Students t-test. Both quantification methods demonstrated a correlation between age and collagen orientation but only with the addition of the Gabor filter was the correlation significant in both the papillary and reticular dermis. Overall, with the Gabor filter the reticular dermis has a higher correlation co-efficient (R^2^ = 0.956) compared to papillary dermis (R^2^ = 0.920) suggesting that the latter compartment is slightly more resilient to age-related damage.

### Loss of structure in diabetic skin

**Figure 5:**
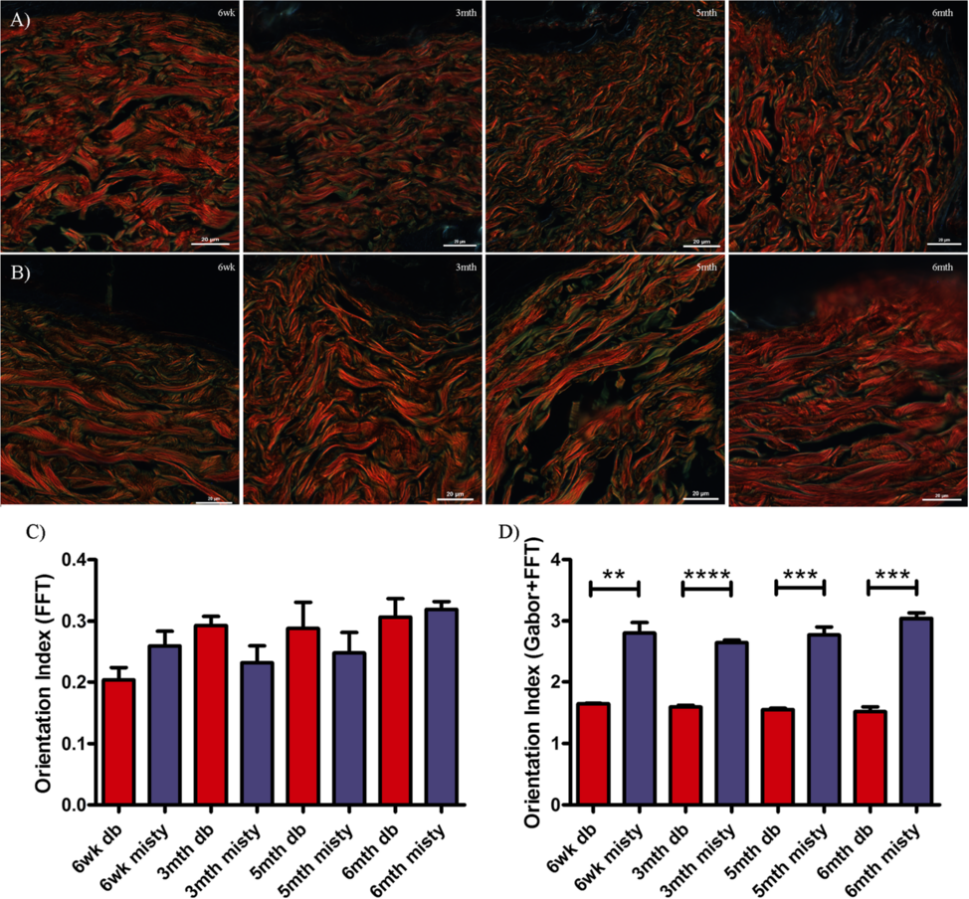
**Dermal collagen organisation in the diabetic mouse.** Representative images of **A)** Misty and **B)***db/db* diabetic skin from mice at 6wk, 3mth, 5mth and 6mth stained with picrosirius. Quantification using the Gabor/ FFT methodology **(C)** or by FFT alone **(D)**, the bar equates to the mean and hair lines are standard error of the mean (S.E.M.). Students’ t-test was used to compare diabetic and lean mice at each time-point, and time course comparisons within *db/db* and Misty groups were compared with a one way ANOVA and a Dunnett’s post-hoc analysis using 6wk old animals as the reference group: ** p < 0.01; *** p < 0.001 (n > 3 animals per group, original magnification 90X, scale bar = 20 μm in each case).

Diabetes has many cutaneous sequelae, with the vast majority of individuals suffering from type 2 diabetes experiencing skin complications during the natural history of their disease [28]. Problems range from the sub-clinical, such as collagen alteration, to potentially catastrophic events, most notably delayed wound healing. Mice lacking the receptor for the satiety regulator leptin are hyperphagic and demonstrate increased post-natal weight gain, which promotes hyperinsulinaemia and eventually hyperglycaemia [29]. We have previously reported a dramatic decrease in dermal depth in these mice [10], which was associated with a qualitative change in collagen fibre orientation. These parallel some of the changes reported in human ageing [8]. Therefore, we also chose to assess collagen basketweave in picrosirius-stained dorsal skin samples from *db/db* mice (a model of type 2 diabetes meillitus) compared lean WT animals at various ages (Figure 5).

Analysis of picrosirius stained skin by FFT alone showed a decrease in basketweave integrity, as defined by an increase in the Orientation Index, between 6wk and 3mth (by which time mice were hyperglycaemic). After 3mths, this analysis suggested that no further loss of structure occurred. However, no statistically significant differences in dermal integrity discriminated diabetic and lean mouse skin at any time point (Figure 5C). Application of the Gabor filter revealed a progressive loss in dermal integrity, and the degradation seen by 6mth was consistent with the loss of cutaneous integrity that one would anticipate in diabetic animals. More importantly, inclusion of the Gabor filter allowed discrimination between lean and diabetic skin structure at each time point (Figure 5D). Interestingly, Misty skin appeared to have a more ordered basketweave structure compared to the C57Bl6 mice and this unexpected strain variation is a current avenue of investigation in our laboratory.

Our method also allowed us to determine subtle changes in *db/db* skin structure with age. The collagen basketweave structure decreased in organisation by −3.1% (3mth; p = 0.1372), -5.9% (5mth; p = 0.0176) and −7.8% (6mth; p = 0.1833) compared to 6wk. Furthermore, we found that the orientation indices calculated from the Gabor plus Fourier method exhibited a significant inverse correlation with increasing age of diabetic skin (R^2^ = 0.9936) but not with in the control skin (R^2^ = 0.3737), whilst no correlation or significant differences between ages were observed with the Fourier alone method (Table 2).

**Table 2:** Diabetic correlation co-efficient calculations

	***db/db *gabor**	**Misty gabor**	***db/db *FFT**	**Misty FFT**
95% CI	−0.9999 to −0.8511	−0.8481 to 0.9905	−0.6437 to 0.9964	−0.8531 to 0.9901
P value	0.0032	0.3887	0.1676	0.4002
R^2^	0.9936	0.3737	0.6929	0.3598

## Discussion

Image analysis techniques that exploit the frequency domain are attractive as they generate spectra informed by texture. We have developed a methodology that can quantify the organised structure or texture within images. Due to the highly-organised basketweave conformation of healthy mammalian dermis being lost or at least compromised in pathological conditions, or with chronological age, the investigation of these pathological states should be tractable to image analysis. Indeed the loss of both fibrillar collagens and the well-organised collagen structure with increasing age has long been known [8,9,30], and loss of collagen organization in gross pathological states such as fibrosis (as assessed by FFT methods) were previously documented [6,13,19,31]. However, these are untested with respect to more subtle changes in structure (which still have many pathological sequelae). Moreover, they are also untested in mouse tissue, in which the finer collagen structure is considerably more difficult to discern than in humans. FFT-based quantification of multi-photon microscopic images of mouse dermis has been reported, but this was not designed to specifically assess basketweave, and this technology is expensive and beyond the reach of many laboratories [9]. A wide range of therapeutic and cosmetic interventions target skin structure, texture and function, and hence tools to determine incremental changes in the integrity of the collagen basketweave are needed. These analytical tools would be particularly attractive if they could extract information from images generated by routine or inexpensive laboratory equipment, rather than electron micrographs or confocal imaging.

Simple H&E stained images are informative (particularly if eosin auto-fluorescence is exploited), but they do not reveal collagen basketweave. Cross-polar images of picrosirius-stained histological skin sections are able to reveal collagen architecture, and relevant optics are inexpensive and retro-fit to many standard microscopes. We found that by applying a FFT to cross-polar photomicrographs, we were able to quantify a shift in collagen organisation in extreme age (i.e. in skin from 20mth old mice). However, incipient collagen changes went undetected in samples from one year old mice. To improve sensitivity, we decided to deploy a Gabor filter to improve edge detection prior to application of a FFT. This yields a more complex spectrum, but by quantifying pixel distributions in four planes we were able to create a sensitive collagen orientation index. In this way, we were able to detect subtle changes in collagen that were not revealed by FFT alone. Further testing revealed that 5° rotations of the images used in this study still facilitated discrimination between biological groups, although larger rotations that take the basketweave out of phase with the Gabor filter (i.e. 20-30°) result in a loss of sensitivity (not shown). This suggests that to ensure optimal performance of the algorithm, all images should be orientated in the same plane, as far as possible. Overall, our improved method enabled us to assess subtle age-related differences in the sub-compartments of the dermis and, more importantly, to quantify collagen damage in models of diabetes.

## Conclusions

Our improved measurement of texture and divergence from regular structure in multiple planes provides superior measurement of collagen orientation in skin and thus is widely applicable to dermatological research. In addition, the combination of the Gabor filter and FFT is likely to have utility beyond the quantification of texture in skin and biological imaging as the fundamental principle of measuring divergence from a regular shape is of wider utility across scientific and mathematical disciplines.

### Availability

A fully functioning version with example images and full instructions for our analysis platform is available to download from: http://webspace.buckingham.ac.uk/klanglands/.

## Abbreviations

mth: month; wk: week.

## Competing interests

All authors declare that they have no competing interests.

## Authors’ contributions

OSO developed the image analysis technique and helped to draft the manuscript. JLS captured the images, performed the statistical analysis, created the graphs and drafted the manuscript. PEH performed the histology. CJS and ETW raised and provided the animals for the study. MAC and CJS helped to draft the manuscript. KL and SJ conceived the study, and participated in its design and coordination and helped to draft the manuscript. All authors read and approved the final manuscript.

## References

[B1] McGibbon D Rook‘s textbook of dermatology, 7th edition Clin Exp Dermatol 2006 31 1 178 179 10.1111/j.1365-2230.2005.02034.x

[B2] Kadler KE Baldock C Bella J Boot-Handford RP Collagens at a glance J Cell Sci 2007 120 Pt 12 1955 1958 17550969 10.1242/jcs.03453

[B3] Myllyharju J Kivirikko KI Collagens, modifying enzymes and their mutations in humans, flies and worms Trends in Genetics: TIG 2004 20 1 33 43 10.1016/j.tig.2003.11.004 14698617

[B4] Meigel WN Gay S Weber L Dermal architecture and collagen type distribution Arch Dermatol Res 1977 259 1 1 10 10.1007/BF00562732 71020

[B5] Sorrell JM Caplan AI Fibroblast heterogeneity: more than skin deep J Cell Sci 2004 117 Pt 5 667 675 14754903 10.1242/jcs.01005

[B6] van Zuijlen PP Ruurda JJ van Veen HA van Marle J van Trier AJ Groenevelt F Kreis RW Middelkoop E Collagen morphology in human skin and scar tissue: no adaptations in response to mechanical loading at joints Burns 2003 29 5 423 431 10.1016/S0305-4179(03)00052-4 12880721

[B7] Rawlins JM Lam WL Karoo RO Naylor IL Sharpe DT Quantifying collagen type in mature burn scars: a novel approach using histology and digital image analysis J Burn Care Res 2006 27 1 60 65 10.1097/01.bcr.0000192266.14329.7b 16566538

[B8] Varani J Warner RL Gharaee-Kermani M Phan SH Kang S Chung JH Wang ZQ Datta SC Fisher GJ Voorhees JJ Vitamin A antagonizes decreased cell growth and elevated collagen-degrading matrix metalloproteinases and stimulates collagen accumulation in naturally aged human skin J Invest Dermatol 2000 114 3 480 486 10.1046/j.1523-1747.2000.00902.x 10692106

[B9] Wu S Li H Yang H Zhang X Li Z Xu S Quantitative analysis on collagen morphology in aging skin based on multiphoton microscopy J Biomed Opt 2011 16 4 040502 10.1117/1.3565439 21529064

[B10] Al-Habian AZ MS Stocker CJ Kepczynska MA Wargent ET Cawthorne MA Langlands K Abstract: Increasing insulin resistance correlates with progressive skin damage in murine models of obesity and diabetes J Investig Dermatol 2011 131 S1 S34

[B11] Har-Shai Y Amar M Sabo E Intralesional cryotherapy for enhancing the involution of hypertrophic scars and keloids Plast Reconstr Surg 2003 111 6 1841 1852 10.1097/01.PRS.0000056868.42679.05 12711943

[B12] Har-Shai Y Sabo E Rohde E Hyams M Assaf C Zouboulis CC Intralesional cryosurgery enhances the involution of recalcitrant auricular keloids: a new clinical approach supported by experimental studies Wound Repair Regen 2006 14 1 18 27 10.1111/j.1524-475X.2005.00084.x 16476068

[B13] Khorasani H Zheng Z Nguyen C Zara J Zhang X Wang J Ting K Soo C A quantitative approach to scar analysis Am J Surg Pathol 2011 178 2 621 628 10.1016/j.ajpath.2010.10.019 PMC3070584 21281794

[B14] de Vries HJ Enomoto DN van Marle J van Zuijlen PP Mekkes JR Bos JD Dermal organization in scleroderma: the fast Fourier transform and the laser scatter method objectify fibrosis in nonlesional as well as lesional skin Lab Invest 2000 80 8 1281 1289 10.1038/labinvest.3780136 10950119

[B15] Vaezy S Smith LT Milaninia A Clark JI Two-dimensional fourier analysis of electron micrographs of human skin for quantification of the collagen fiber organization in the dermis J Electron Microsc (Tokyo) 1995 44 5 358 364 8568449

[B16] Noorlander ML Melis P Jonker A Van Noorden CJ A quantitative method to determine the orientation of collagen fibers in the dermis J Histochem Cytochem 2002 50 11 1469 1474 10.1177/002215540205001106 12417612

[B17] van Zuijlen PP de Vries HJ Lamme EN Coppens JE van Marle J Kreis RW Middelkoop E Morphometry of dermal collagen orientation by Fourier analysis is superior to multi-observer assessment J Pathol 2002 198 3 284 291 10.1002/path.1219 12375260

[B18] van Zuijlen PP Lamme EN van Galen MJ van Marle J Kreis RW Middelkoop E Long-term results of a clinical trial on dermal substitution. A light microscopy and Fourier analysis based evaluation Burns 2002 28 2 151 160 10.1016/S0305-4179(01)00085-7 11900939

[B19] Verhaegen PD Marle JV Kuehne A Schouten HJ Gaffney EA Maini PK Middelkoop E Zuijlen PP Collagen bundle morphometry in skin and scar tissue: a novel distance mapping method provides superior measurements compared to Fourier analysis J Microsc 2012 245 1 82 89 10.1111/j.1365-2818.2011.03547.x 21919907

[B20] Daugman J How iris recognition works. Circuits and Systems for Video Technology IEEE Trans 2004 14 1 21 30

[B21] Daugman JG Complete discrete 2-D Gabor transforms by neural networks for image analysis and compression. Acoustics, Speech and Signal Processing IEEE Trans 1988 36 7 1169 1179

[B22] Junqueira LC Bignolas G Brentani RR Picrosirius staining plus polarization microscopy, a specific method for collagen detection in tissue sections Histochem J 1979 11 4 447 455 10.1007/BF01002772 91593

[B23] Sweat F Puchtler H Rosenthal SI Sirius red F3BA as a stain for connective tissue Arch Pathol 1964 78 69 72 14150734

[B24] Puchtler H Waldrop FS Valentine LS Polarization microscopic studies of connective tissue stained with picro-sirius red FBA Beitr Pathol 1973 150 2 174 187 10.1016/S0005-8165(73)80016-2 4129194

[B25] Mays PK McAnulty RJ Campa JS Laurent GJ Age-related changes in collagen synthesis and degradation in rat tissues. Importance of degradation of newly synthesized collagen in regulating collagen production Biochem J 1991 276 Pt 2 307 313 2049064 10.1042/bj2760307 PMC1151092

[B26] Fornieri C Quaglino D Jr Mori G Correlations between age and rat dermis modifications. Ultrastructural-morphometric evaluations and lysyl oxidase activity Aging (Milano) 1989 1 2 127 138 2577359 10.1007/BF03323883

[B27] Boyer B Kern P Fourtanier A Labat-Robert J Age-dependent variations of the biosyntheses of fibronectin and fibrous collagens in mouse skin Exp Gerontol 1991 26 4 375 383 10.1016/0531-5565(91)90049-R 1936196

[B28] Romano G Moretti G Di Benedetto A Giofre C Di Cesare E Russo G Califano L Cucinotta D Skin lesions in diabetes mellitus: prevalence and clinical correlations Diabetes Res Clin Pract 1998 39 2 101 106 10.1016/S0168-8227(97)00119-8 9597379

[B29] Hummel KP Dickie MM Coleman DL Diabetes, a new mutation in the mouse Science 1966 153 3740 1127 1128 10.1126/science.153.3740.1127 5918576

[B30] Varani J Dame MK Rittie L Fligiel SE Kang S Fisher GJ Voorhees JJ Decreased collagen production in chronologically aged skin: roles of age-dependent alteration in fibroblast function and defective mechanical stimulation Am J Pathol 2006 168 6 1861 1868 10.2353/ajpath.2006.051302 16723701 PMC1606623

[B31] van Zuijlen PP Angeles AP Kreis RW Bos KE Middelkoop E Scar assessment tools: implications for current research Plast Reconstr Surg 2002 109 3 1108 1122 10.1097/00006534-200203000-00052 11884845

